# Analysis of Geometric and Hemodynamic Profiles in Rat Arteriovenous Fistula Following PDE5A Inhibition

**DOI:** 10.3389/fbioe.2021.779043

**Published:** 2021-12-02

**Authors:** Hannah Northrup, Maheshika Somarathna, Savanna Corless, Isabelle Falzon, John Totenhagen, Timmy Lee, Yan-Ting Shiu

**Affiliations:** ^1^ Department of Biomedical Engineering, University of Utah, Salt Lake City, UT, United States; ^2^ Division of Nephrology and Hypertension, University of Utah Department of Internal Medicine, Salt Lake City, UT, United States; ^3^ Division of Nephrology, Department of Medicine, University of Alabama at Birmingham, Birmingham, AL, United States; ^4^ Department of Radiology, University of Alabama at Birmingham, Birmingham, AL, United States; ^5^ Veterans Affairs Medical Center, Birmingham, AL, United States; ^6^ Veterans Affairs Medical Center, Salt Lake City, UT, United States

**Keywords:** arteriovenous fistula, computational fluid dynamics, shear stress, outward remodeling, vessel dilation

## Abstract

Arteriovenous fistula (AVF) is essential for chronic kidney disease (CKD) patients on hemodialysis, but treatment for AVF maturation failure remains an unmet clinical need. Successful AVF remodeling occurs through sufficient lumen expansion to increase AVF blood flow and lumen area. Aberrant blood flow is thought to impair AVF remodeling, but previous literature has largely focused on hemodynamics averaged over the entire AVF or at a single location. We hypothesized that hemodynamics is heterogeneous, and thus any treatment’s effect size is heterogeneous in the AVF. To test our hypothesis, we used the PDE5A inhibitor sildenafil to treat AVFs in a rat model and performed magnetic resonance imaging (MRI) based computational fluid dynamics (CFD) to generate a detailed spatial profile of hemodynamics in AVFs. 90 mg/kg of sildenafil was administered to rats in their drinking water for 14 days. On day 14 femoral AVFs were created in rats and sildenafil treatment continued for another 21 days. 21 days post-AVF creation, rats underwent non-contrast MRI for CFD and geometrical analysis. Lumen cross-sectional area (CSA) and flow rate were used to quantify AVF remodeling. Parameters used to describe aberrant blood flow include velocity magnitude, wall shear stress (WSS), oscillatory shear index (OSI), and vorticity. Geometrical parameters include arterial-venous (A-V) distance, anastomosis angle, tortuosity, and nonplanarity angle magnitude. When averaged across the entire AVF, sildenafil treated rats had significantly higher CSA, flow rate, velocity, WSS, OSI, and vorticity than control rats. To analyze heterogeneity, the vein was separated into zones: 0–5, 5–10, 10–15, and 15–20 mm from the anastomosis. In both groups: 1) CSA increased from the 0–5 to 15–20 zone; 2) velocity, WSS, and vorticity were highest in the 0–5 zone and dropped significantly thereafter; and 3) OSI increased at the 5–10 zone and then decreased gradually. Thus, the effect size of sildenafil on AVF remodeling and the relationship between hemodynamics and AVF remodeling depend on location. There was no significant difference between control and sildenafil groups for the other geometric parameters. Rats tolerated sildenafil treatment well, and our results suggest that sildenafil may be a safe and effective therapy for AVF maturation.

## Introduction

Maturation failure of the arteriovenous fistula (AVF) remains a significant problem for chronic kidney disease patients on hemodialysis, with failure rates as high as 60% (Dember et al., 2008; Cheung et al., 2017). AVF maturation requires remodeling of the vessel wall to sufficiently increase AVF flow rate and lumen area for dialysis. The two leading causes of AVF maturation failure are poor outward remodeling and excessive inward remodeling. Outward remodeling results from the relaxation of vascular smooth muscle cells to dilate the vessel and the remodeling of the vessel wall to stabilize the larger lumen. Inward remodeling results from the formation of neointimal hyperplasia to decrease the open lumen area. Successful AVF remodeling occurs when outward remodeling is more dominant than inward remodeling (Shiu et al., 2019). One potential strategy to achieve sufficient outward remodeling is to increase relaxation of vascular smooth muscle cells (SMCs), allowing the fistula vein to dilate and hence increase lumen area. Other disciplines such as hypertension have used phosphodiesterase 5 (PDE5) inhibitors, e.g. sildenafil, to promote vessel dilation. Although the vein has less SMCs than the artery, Medina et al. reported that sildenafil had a similar relaxant effect on radial arteries and cephalic veins harvested from organ donors (Medina et al., 2000). Additionally, the multicenter Hemodialysis Fistula Maturation Consortium Study found that the endothelium-independent and SMC-dependent capacity of the brachial artery to dilate, assessed by nitroglycerin-mediated dilation, was positively associated with AVF lumen diameter and flow rate at 6 weeks after AVF creation (Allon et al., 2016). Taken together, sildenafil can dilate both the vein and artery, and arterial dilation is important for the development of AVF venous limbs. Thus, we hypothesized that PDE5 inhibitors may improve AVF maturation, as indicated by increased flow rate and cross-sectional lumen area (CSA). We tested this hypothesis by investigating the effect of sildenafil on femoral AVFs in rats using magnetic resonance imaging (MRI)-based computational fluid dynamics (CFD) and geometrical analysis.

CFD simulates the blood flow throughout the AVF, allowing for a detailed investigation of hemodynamics such as wall shear stress (WSS), oscillatory shear index (OSI), and vorticity. In response to physiologically relevant laminar arterial WSS, endothelial cells release vasodilators such as nitric oxide, resulting in flow-mediated dilation (Pyke and Tschakovsky, 2005; Shiu et al., 2019). OSI describes how the WSS vector differs with the time-averaged wall shear stress vector (Lantz et al., 2014) and vorticity quantifies the rotation of the fluid (Pike et al., 2019). We have previously established MRI-CFD protocols to investigate these parameters in AVFs in patients (He et al., 2013) and a mouse model (Pike et al., 2017, 2019). Here we establish a protocol for rats.

Through geometrical analysis, we can analyze the CSA, artery-vein (A-V) distance, anastomosis angle, tortuosity, and nonplanarity angle of an AVF. The A-V distance is the distance between the fistula vein and the proximal artery. The anastomosis angle describes the angle between the fistula vein and proximal artery. Tortuosity describes the twisting of the fistula vein, and nonplanarity angle describes how the fistula vein is aligned with the anastomosis. These parameters may give us further insights into the mechanics of AVF remodeling. These analyses have been performed by us to AVFs in patients (Yang et al., 2020; He et al., 2021) and a mouse model (Falzon et al., 2020). Here we modified these protocols for rats.

It is important to note that hemodynamic and geometric parameters may not be uniform throughout the AVF during its remodeling. For example, the suture at the anastomosis may limit vessel dilation near the anastomosis, the degree of stenosis may vary throughout the vein, and the anatomy of the animal species may impact the hemodynamics and geometry at various locations along the fistula vein. Averaged values may lose key information locally. Likewise, data extracted from a single location in the vessel do not capture key information from the surrounding areas. Therefore, in this study, we investigated how the geometry and hemodynamics vary by location throughout the fistula vein, using the arteriovenous anastomosis as the landmark. We hypothesized that hemodynamics is not uniform through the fistula vein, and thus any treatment’s effect size is also not uniform throughout the AVF.

## Materials and Methods

### Animal Procedure: Sildenafil Treatment, AVF Creation and Magnetic Resonance Imaging

**FIGURE 1:**
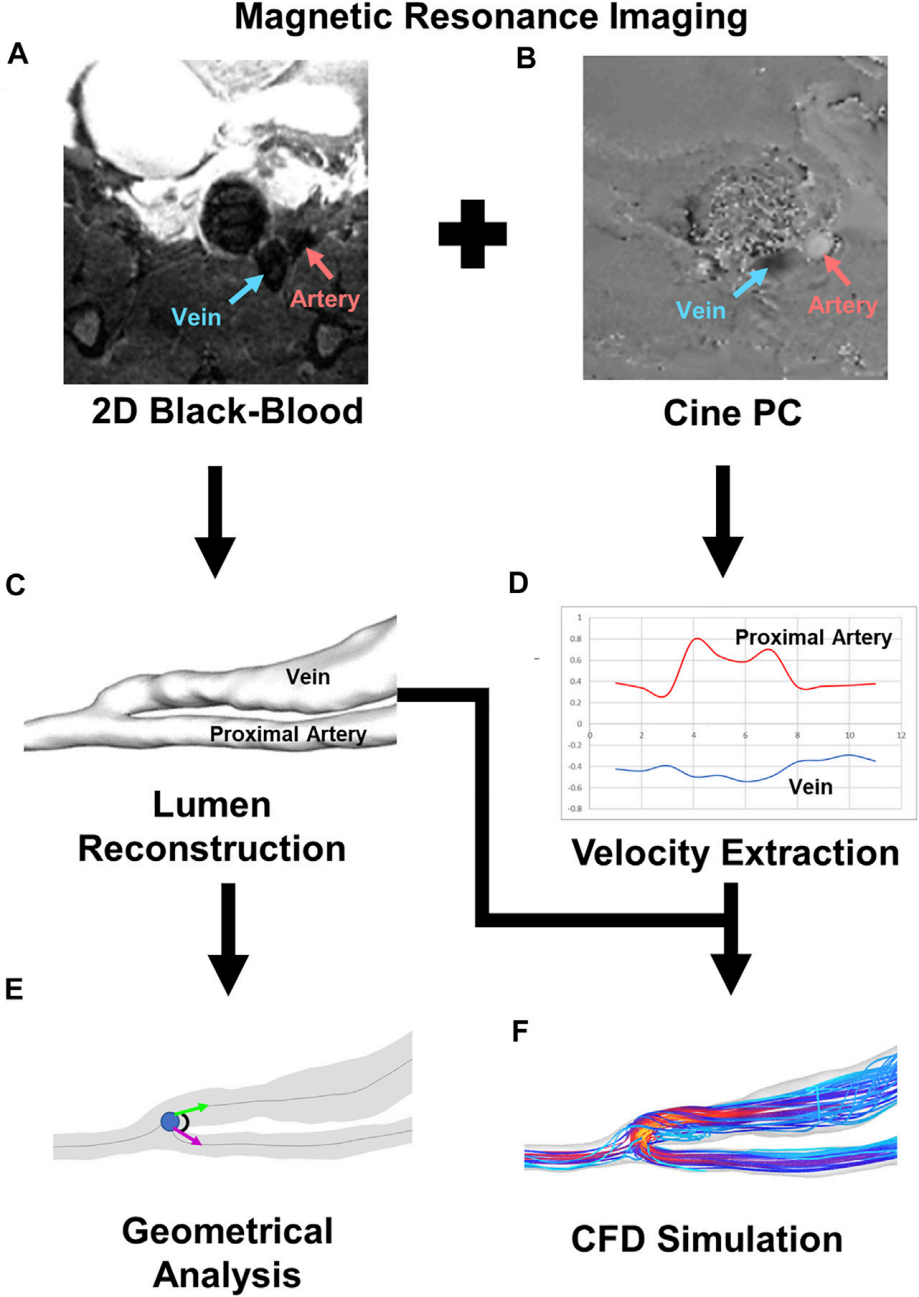
Pipeline for CFD and geometrical analysis. **(A)** 2D black-blood MRI image for AVF lumen geometry visualization. Red and blue arrows point to the artery and vein, respectively. **(B)** Cine-phase contrast image for velocity mapping. Red and blue arrows point to the artery and vein, respectively. **(C)** Representative image of lumen reconstruction created from black-blood MRI. **(D)** Representative graph of velocity extracted from cine-phase contrast image. Red represents the proximal artery, and blue represents the fistula vein. **(E)** Representative image of geometrical analysis for anastomosis angle. **(F)** Representative image of velocity streamlines from CFD simulation.

All animal studies and experiments were approved by the University of Alabama at Birmingham Institutional Animal Care and Use Committee and were performed in accordance with National Institute of Health guidelines. Sildenafil was administered daily to 12–16 week-old male Sprague-Dawley rats at 90 mg/kg in drinking water. 14 days after starting sildenafil treatment, femoral vein (end) to femoral artery (side) AVFs were surgically created. Sildenafil treatment was continued in rats for 21 days post-AVF creation, at which time animals underwent non-contrast magnetic resonance imaging (MRI) performed by a 9.4 T Bruker BioSpec 94/20 MRI machine (Bruker Biospin, Billerica, MA) as described in Table 1. Briefly, rats were imaged with time-of-flight, T2-black-blood, and cardiac-gated velocity mapping (Figure 1). Rats were positioned in the supine position, and a 24 mm surface coil was centered between the knees and hips. 11 frames were collected for velocity mapping with VENC of 120 cm/s and 15 ms between frames using respiration gating. 37 contiguous 1-mm thick axial slices with a resolution of 0.2 mm were acquired for black blood imaging.

**TABLE 1:** Contrast-free MRI acquisition protocol. Note: RARE = Rapid Acquisition with Relaxation Enhancement, TR = Repetition time, TE = Echo Time, TOF = Time of Flight.

Scan	Purpose	Imaging parameters
T2-weighted RARE	Scout images to verify correct coil placement and location and orientation of AVF	TR 2000 ms, TE 24 ms, RARE factor 4, 1 average, matrix 256 × 256, FOV 51.2 mm × 51.2 mm, in plane resolution 0.2 mm
TOF	Obtain detailed information on geometry	FLASH method, TR 18 ms, TE 4 ms, 8 averages, matrix 171 × 171, FOV 51.2 mm × 51.2 mm, resolution 0.3 mm, 50 overlapping axial slices at a thickness of 1 and 0.75 mm between slice spacing
T2-weighted fast spin echo with black-blood double inversion	Obtain black blood imaging used for AVF lumen reconstruction	TR 10000 ms, TE 33 ms, 4 averages, matrix 256 × 256, FOV 51.2 mm × 51.2 mm, in plane resolution 0.2 mm
Gradient echo velocity mapping	Obtain blood flow measures used for velocity extraction	TR 15 ms, TE 6 ms, 16 averages, matrix 150 × 256, FOV 30 mm × 51.2 mm, in plane resolution 0.2 mm with single 2 mm thick axial slices

### Geometrical Analysis

**FIGURE 2:**
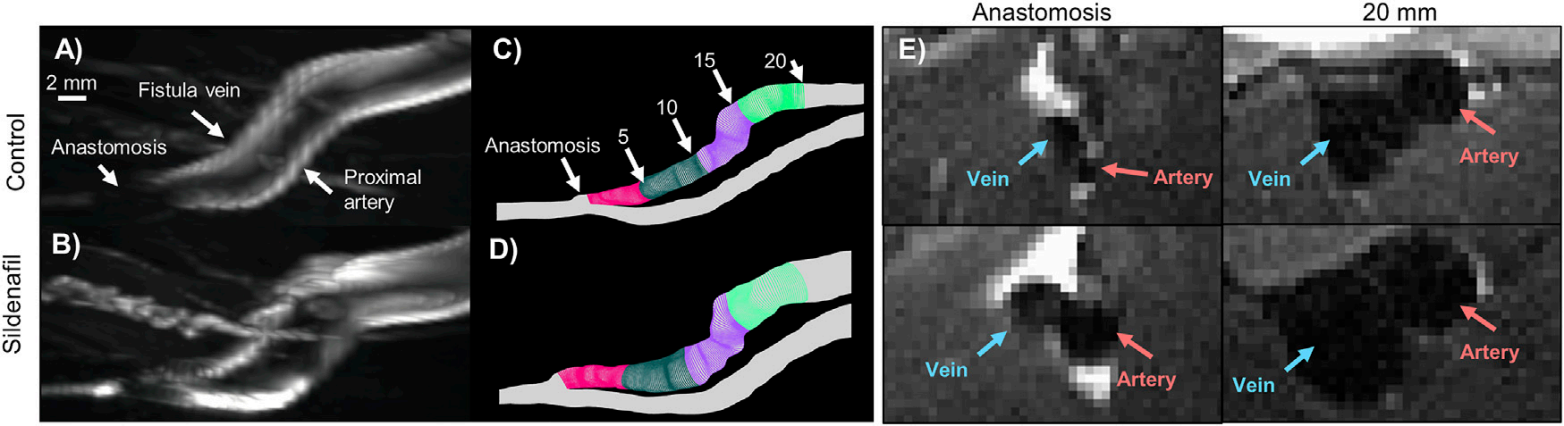
MRI images and lumen geometrical models of femoral AVF for control and sildenafil treated rats. **(A,B)** Maximum intensity projection of femoral AVF in a control rat **(A)** and a sildenafil treated rat **(B)**. **(C,D)** Lumen geometry reconstruction of AVF in a control rat (C) and a sildenafil treated rat (D), with 0–5 mm zone shown in magenta, 5–10 mm zone shown in dark green, 10–15 mm zone in purple, and 15–20 mm zone in light green. **(E)** Black-blood MRI images of the anastomosis and a slice 20 mm away from the anastomosis for control and sildenafil treated rats. Red and blue arrows point to the artery and vein, respectively.

**FIGURE 3:**
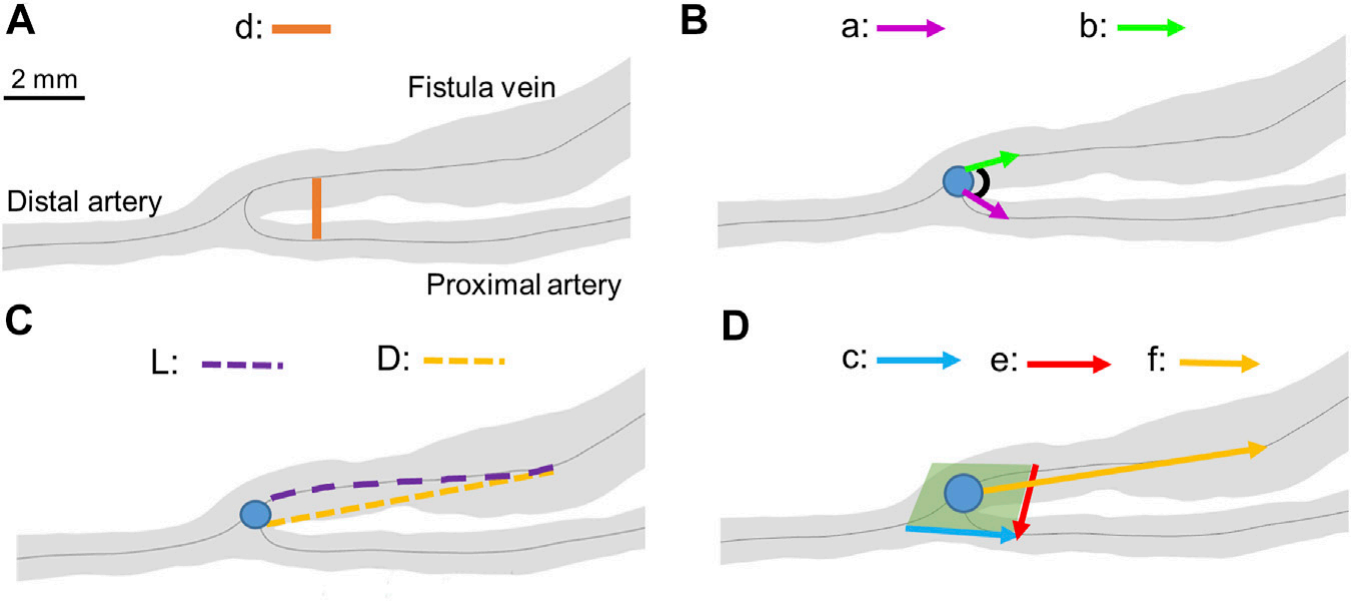
Geometrical parameter calculations. **(A)** A-V distance. “d” represents the distance between the proximal artery and the fistula vein. **(B)** Anastomosis angle. “a” represents vector from the anastomosis origin (blue dot) to a point 2 mm away in the proximal artery. “b” represents the vector from the anastomosis origin to a point 2 mm away in the fistula vein. **(C)** Tortuosity. “L” represents the curved length of the centerline to a point 6.5 mm away from the anastomosis origin. “D” represents the straight-line length to a point 6.5 mm away from the anastomosis origin. **(D)** Nonplanarity angle. The green plane represents the plane created from vectors “c” and “e”. “f” represents the vector from the anastomosis angle to a point 6.5 mm away in the fistula vein. The blue dots represent the anastomosis origin in all panels.

Geometrical analysis was performed as previously described for mouse and human (Falzon et al., 2020; He et al., 2021) with modifications for rat. First, Amira (Thermo Fisher Scientific, Waltham, Mass) was used to reconstruct the lumen from the 2D black-blood MRI sequence. Using Amira’s segmentation editor, the lumen was selected, and then the surface generated (Figure 2). The lumen was smoothed at 60 iterations and a lambda of 0.6. The reconstructed lumen was exported as an STL ASCII format to be used in the Vascular Modeling Toolkit (VMTK) (available at: www.vmtk.org). In VMTK, centerlines were calculated with points at 0.1 mm intervals starting at the anastomosis for the fistula vein, proximal artery, and distal artery. The point of divergence from the distal artery into the proximal artery and fistula vein was calculated as the anastomosis origin. The centerlines and STL of the reconstruction were used in MATLAB (MATLAB, Natick, Mass) to calculate the CSA at 0.1 mm intervals along the centerline (He et al., 2013). The x, y, and z coordinates of the centerlines and anastomosis origin were used to calculate the geometric parameters of A-V distance, anastomosis angle, tortuosity, and nonplanarity angle, as previously described for human and mouse, with modifications for rat (Figure 3; Eqs 1–4) (Falzon et al., 2020; He et al., 2021).

A-V distance is the distance between the proximal artery and the fistula vein. It was calculated at each centerline point along the proximal artery. The corresponding fistula vein point was determined by creating a cross-section of the AVF at a right angle to the proximal artery in Amira. The corresponding fistula vein point was the point of the AVF that fell within that cross-section. Eq. 1 was used to calculate the distance between the two centerline points.

Anastomosis angle is the angle at the anastomosis origin between the proximal artery and fistula vein. Anastomosis angle was calculated as described in Eq. 2, where a and b are straight-line vectors starting at the anastomosis origin and ending 2 mm away in the fistula vein and proximal artery, respectively.

Tortuosity describes the twisting of the fistula vein and was calculated using Eq. 3. L is the curved length of the centerline to a point 6.5 mm away from the anastomosis origin, and D is the straight-line length.

Nonplanarity angle describes how close the fistula vein falls to a plane created at the anastomosis. It was calculated using Eq. 4, where c and e are vectors at 2 mm away from the anastomosis origin in the proximal artery and fistula vein, respectively, and f is a straight-line vector 6.5 mm away from the anastomosis origin on the fistula vein. The absolute value of the nonplanarity angle was reported as the nonplanarity angle magnitude.

The values of a, b, c, e, and f were chosen by testing various values for each. The values that resulted in the median anastomosis angle, tortuosity, and nonplanarity angle were selected. The value of 6.5 mm was selected for D, and f. 2 mm was selected for a and b. Analysis of the various vectors and distances can be found in the supplemental material (Supplementary Figure S1).

$$\begin{array}{c} A-V\;Dis\tan ce={\left({\left(x_{vein}-x_{artery}\right)}^{2}+{\left(y_{vein}-y_{artery}\right)}^{2}+{\left(z_{vein}-z_{artery}\right)}^{2}\right)}^{\frac{1}{2}} \end{array}$$ (1)

$$\begin{array}{c} Anastomosis\;Angle=57.3\cos^{-1}\frac{a\cdot b}{\left|a\right|\left|b\right|} \end{array}$$ (2)

$$\begin{array}{c} Tortuosity=\;\frac{L}{D}-1\; \end{array}$$ (3)

$$\begin{array}{c} Nonplanarity\;Angle=57.3\sin^{-1}\frac{\left(c\times e\right)\cdot f}{\left|c\times e\right|\left|f\right|} \end{array}$$ (4)

### CFD Modeling

Computational fluid dynamics was performed as previously described with modifications (He et al., 2013; Pike et al., 2017, 2019) (Figure 1). The lumen was reconstructed, smoothed, and exported in Amira as described in *Geometrical Analysis*. VMTK was used to add flow extensions to prevent entrance effects in the region of interest. The extended geometry was meshed in ANSYS ICEM CFD 2019 R3 (Ansys, Inc., Canonsburg, PA). Approximately 1.5 × 10^6^ tetrahedra in the lumen domain with 4 prism layers at the wall were used for all simulations based on previous mesh independence studies (Pike et al., 2017).

The 2D gradient echo velocity mapping scans were used with ImageJ (available at: https://imagej.nih.gov/ij/) to extract the velocity at the proximal artery and fistula vein for each rat.

ANSYS Fluent 2019 R3 (Ansys, Inc., Canonsburg, PA) was used to run transient CFD simulations assuming a rigid vessel wall with no-slip conditions at the wall. Boundary conditions were taken from the velocity extraction described above and set as a velocity inlet to their respective vessels. The distal artery was set as a pressure outlet with 0 gauge pressure. Blood flow was assumed as laminar, Newtonian, and incompressible. Blood density was prescribed as 1,050 kg/m^3^ with a viscosity of 0.0035 Pa s. Solution methods were set to use the SIMPLE scheme with spatial discretization to Least Squares Cell Based for gradient, Second Order for Pressure, and Second Order Upwind for Momentum. Second Order Implicit was used for the transient formulation. All residuals were set to a convergence criterion of 1 × 10^−5^. The time step size was set to 0.0015 s or a 10th of the time step from the velocity boundary condition. All simulations were run for three cardiac cycles with data from the third cardiac cycle analyzed. Time step size independence was performed by analyzing data extracted from rat CFD simulations run with a 0.015, .0015, and 0.00015 s time step and extracted from the first, second, and third cardiac cycle (Supplementary Figure S2).

### Post-Processing for Hemodynamics

Results were analyzed using Tecplot 360 (Tecplot, Bellevue, Wa) as previously described (Pike et al., 2017, 2019). First, hemodynamic parameters of velocity magnitude, wall shear stress (WSS), oscillatory shear stress (OSI), and vorticity were calculated as described in Eqs 5–8. These parameters were averaged in Tecplot over the cardiac cycle.

$$\begin{array}{c} Velocity={\left(u_{x}^{2}+u_{y}^{2}+u_{z}^{2}\right)}^{\frac{1}{2}} \end{array}$$ (5)

$$\begin{array}{c} WSS={\left(\tau_{w,x}^{2}+\tau_{w,y}^{2}+\tau_{w,z}^{2}\right)}^{\frac{1}{2}} \end{array}$$ (6)

$$\begin{array}{c} OSI=0.5\left(1-\frac{\left|\int_{o}^{t}\tau_{w}dt\right|}{\int_{o}^{t}\left|\tau_{w}\right|dt}\right) \end{array}$$ (7)

$$\begin{array}{c} Vorticity=\nabla \times u \end{array}$$ (8)

The centerlines described in *Geometrical Analysis* were used in MATLAB to calculate points normal to the centerlines. The centerline and centerline normal values were used in Tecplot to create circumferential slices normal to the centerline at 0.1 mm intervals. Results were extracted from each slice and analyzed in Graphpad Prism (GraphPad Software, San Diego, CA) as described below. The flow rate was calculated by multiplying the velocity magnitude from each slice with the corresponding CSA taken from MATLAB.

### Statistics

Statistics were computed in GraphPad Prism. Inter-user variability of the cross-sectional area was calculated by paired *t*-test for data sets that followed a normal distribution, and Wilcoxon signed-rank test used for data that did not follow a normal distribution. CSA, A-V distance, velocity magnitude, flow rate, WSS, OSI, and vorticity data were separated into 5 zones, based on centerline length from the anastomosis: 0–5, 5–10, 10–15, and 15–20 mm (Figures 2C,D), each zone having 50 slices. Data averaged over 0–20 mm are also presented. Unpaired t-tests were used for data that followed a normal distribution. A Mann’s Whitney test was used for data that did not follow a normal distribution. Correlation was determined using Pearson’s correlation test. Data were considered significant if *p* < 0.05 for each analysis. Data contained results from 4 control rats and 4 sildenafil treated rats.

## Results

### AVF Lumen Reconstruction, Area, and Other Geometric Parameters

The AVF lumen segmentation and subsequent reconstruction steps are important because the reconstruction is used to calculate CSA (which is an AVF remodeling outcome) and perform CFD. Reproducibility of the CSA results was analyzed through inter-user variability (Supplementary Figure S3). There was no significant difference between user 1 and user 2 for either sildenafil treated or control rats on average or in any zone.

**FIGURE 4:**
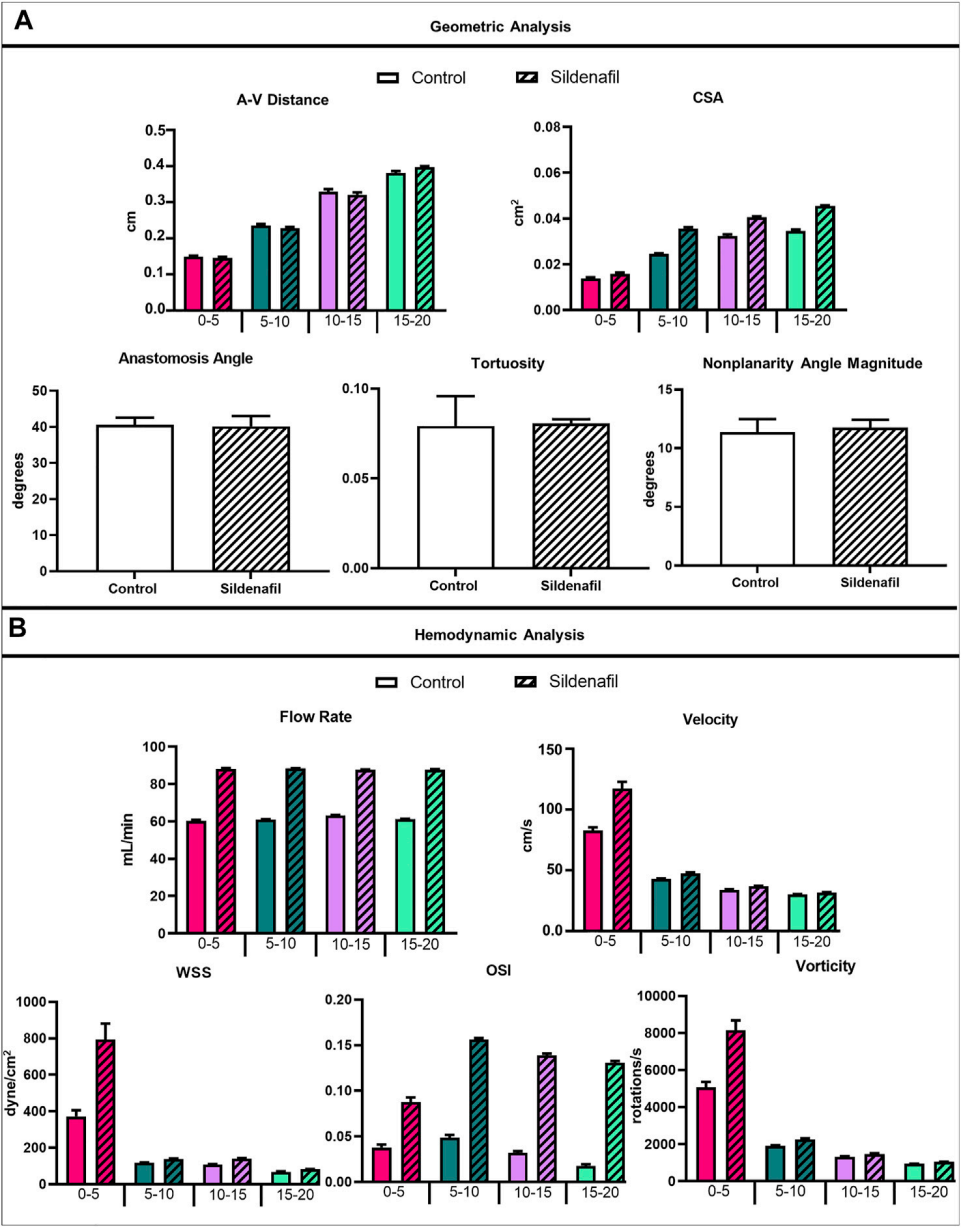
Geometrical and hemodynamic results. **(A)** Geometric analysis results for A-V distance, CSA, anastomosis angle, tortuosity, and nonplanarity angle magnitude. **(B)** Hemodynamic analysis results for flow rate, velocity, WSS, OSI, and vorticity. Data expressed as mean ± SEM. Statistical significance not shown here but described in text. N = 4 for each group.

The AVF CSA averaged over the entire AVF was 0.026 ± 0.009 cm^2^ and 0.034 ± 0.01 cm^2^ for control and sildenafil treated rats, respectively, and sildenafil caused a statistically significant 31% increase. However, the CSA is not uniform, being smaller closer to the anastomosis. As shown in Figure 4A, for the 0–5, 5–10, 10–15, and 15–20 zones, control rats had a mean CSA of 0.014 ± 0.0004 cm^2^, 0.025 ± 0.001 cm^2^, 0.032 ± 0.005 cm^2^, and 0.035 ± 0.004 cm^2^ respectively, whereas sildenafil treated rats had a mean CSA of 0.016 ± 0.005, 0.036 ± 0.0007, 0.041 ± 0.0004, and 0.046 ± 0.002 cm^2^ respectively. The regional CSA of AVF in Sildenafil treated rats was significantly higher than controls within every zone (*p* < 0.05), and the effect size is bigger when further downstream from the anastomosis. Indeed, as shown in the MRI images in Figure 2E, the AVF CSA in sildenafil treated rat is larger than in control rat, and this increase is bigger at 20 mm (a 31% increase) than at the anastomosis (a 14% increase).

The A-V distance averaged over the entire AVF is not statistically different between control (2.73 ± 0.96 cm) and sildenafil (2.73 ± 0.99 cm) rats. However, the local A-V distance is not uniform, being smaller when closer to the anastomosis and increasing significantly when away from the anastomosis. As shown in Figure 4A, for the 0–5, 5–10, 10–15, and 15–20 zones, the A-V distance in control rats was 1.50 ± 0.16, 2.35 ± 0.28, 3.29 ± 0.54, and 3.81 ± 0.35 cm respectively, whereas the A-V distance in sildenafil treated rats was 1.46 ± 0.17, 2.28 ± 0.24, 3.20 ± 0.46, and 3.98 ± 0.18 cm respectively. This result is parallel to the increase in CSA along the AVF. Thus, the vein and artery centerlines move further apart when the lumen becomes bigger.

The geometric parameters of anastomosis angle (40.61° ± 3.93° for control, 40.14° ± 3.93° for sildenafil), tortuosity (0.079 ± 0.033 for control, 0.081 ± 0.004 for sildenafil), and nonplanarity angle magnitude (11.38° ± 2.21° for control, 11.76° ± 1.32° for sildenafil) showed no statistically significant difference between control and sildenafil treated rats (*p* > 0.05) (Figure 4A). The anastomosis angles of our rat AVFs are similar to human radiocephalic AVFs (∼30–60°) in the literature (Sivanesan et al., 1999; Sadaghianloo et al., 2015; Corbett et al., 2018). Indeed, our recent paper (He et al., 2021) found that patients with matured forearm fistulas had an average anastomosis angle of 57°. Additionally, He et al. found that matured forearm fistulas had a tortuosity of 0.081 and had a nonplanarity angle of 17° (He et al., 2021). Our rat model closely resembled the matured forearm fistula for all three parameters of anastomosis angle, tortuosity angle, and nonplanarity angle.

The linear correlation between the following was also analyzed because we found them to be significant in a carotid-jugular mouse AVF model (Falzon et al., 2020): maximum venous CSA and average venous CSA, the minimum venous CSA and maximum A-V distance, tortuosity and maximum A-V distance, vein length at the maximum A-V distance and the maximum A-V distance, the nonplanarity angle magnitude and the maximum A-V distance, and the nonplanarity angle and the vein length at the maximum A-V distance. However, none of the correlations were significant in the femoral rat AVF model (Supplementary Figure S4).

### AVF Hemodynamic Parameters

**FIGURE 5:**
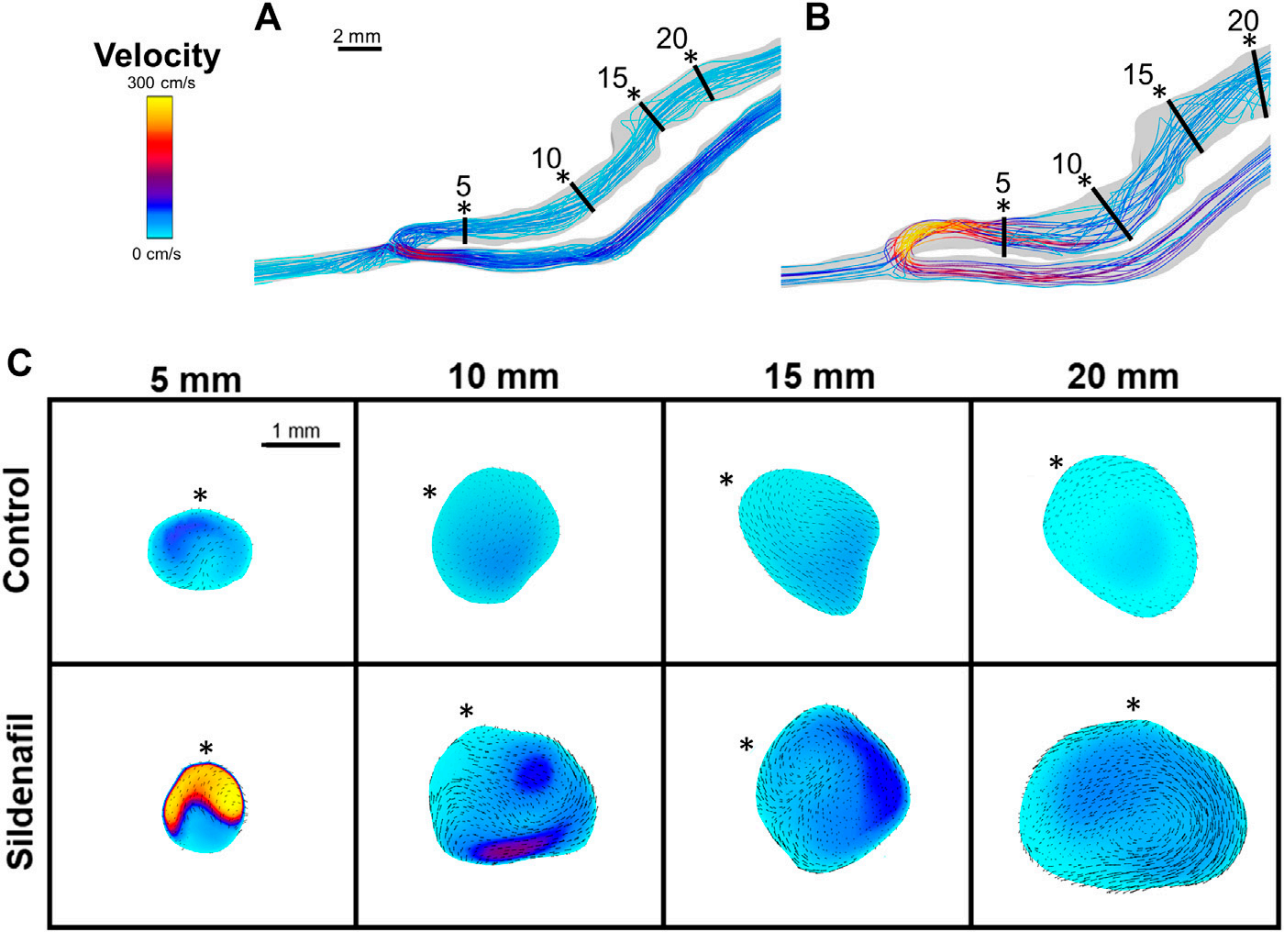
Velocity streamlines and color maps. **(A,B)** Velocity streamlines for AVFs in a control rat (A) and a sildenafil treated rat (B). **(C)** Color maps of velocity magnitude for control (top) and sildenafil (bottom) treated rats at 5, 10, 15, and 20 mm away from the anastomosis. Black lines in panels A and B represent the slice locations. * in panel C represents the top of the slice as shown in panels A and B. Color bar applies to A-C. Scale bar in A applies to A-B. Black dots/arrows in **(C)** are velocity vectors.

**FIGURE 6:**
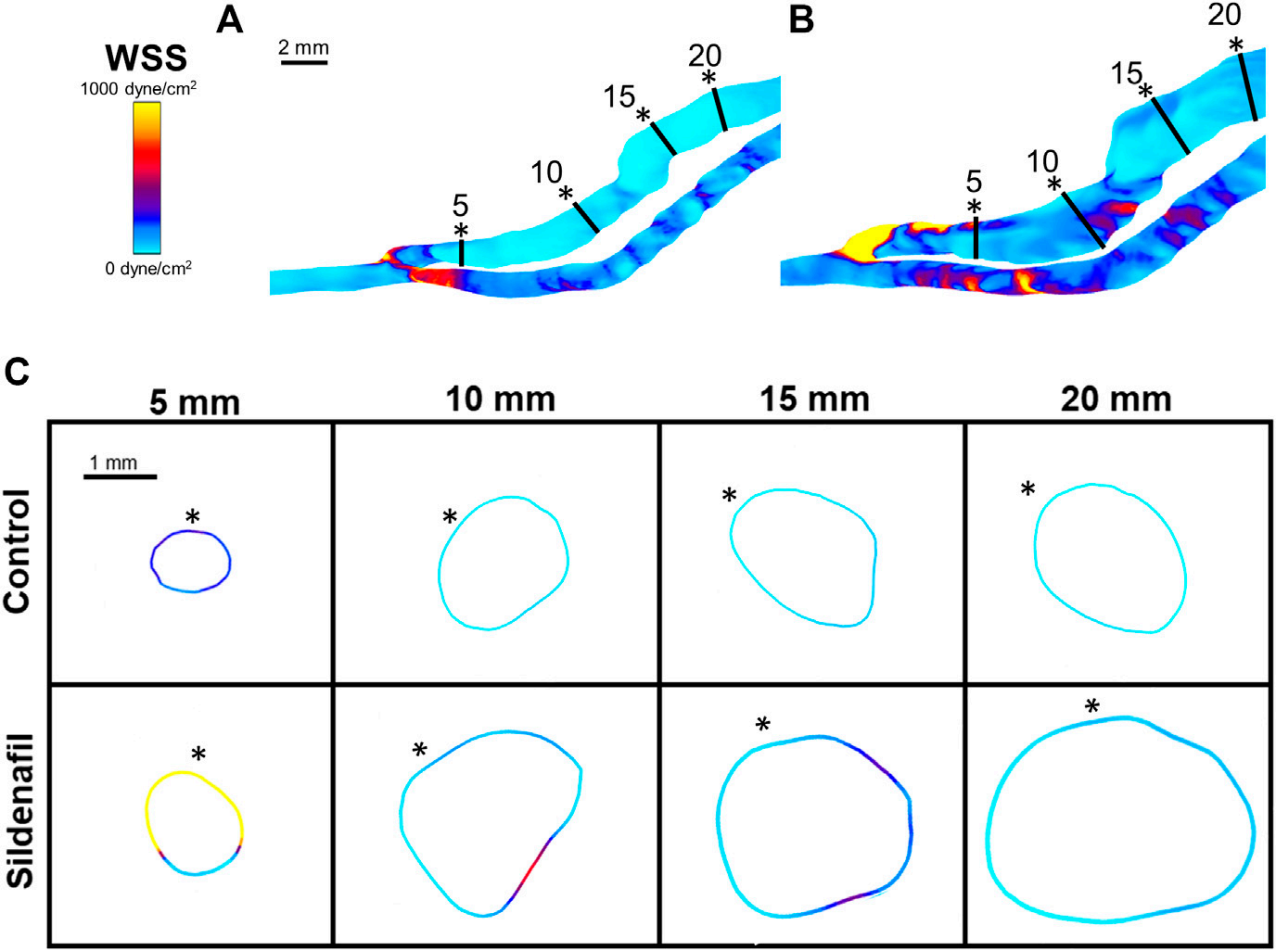
WSS color maps. **(A,B)** WSS for AVFs in a control rat **(A)** and a sildenafil treated rat **(B)**. **(C)** Color maps of WSS for control (top) and sildenafil (bottom) treated rats at 5, 10, 15, and 20 mm away from the anastomosis. Black lines in panels A and B represent the slice locations. * in panel C represents the top of the slice as shown in panels A and B. Color bar applies to **(A–C)**. Scale bar in A applies to **(A–B)**.

**FIGURE 7:**
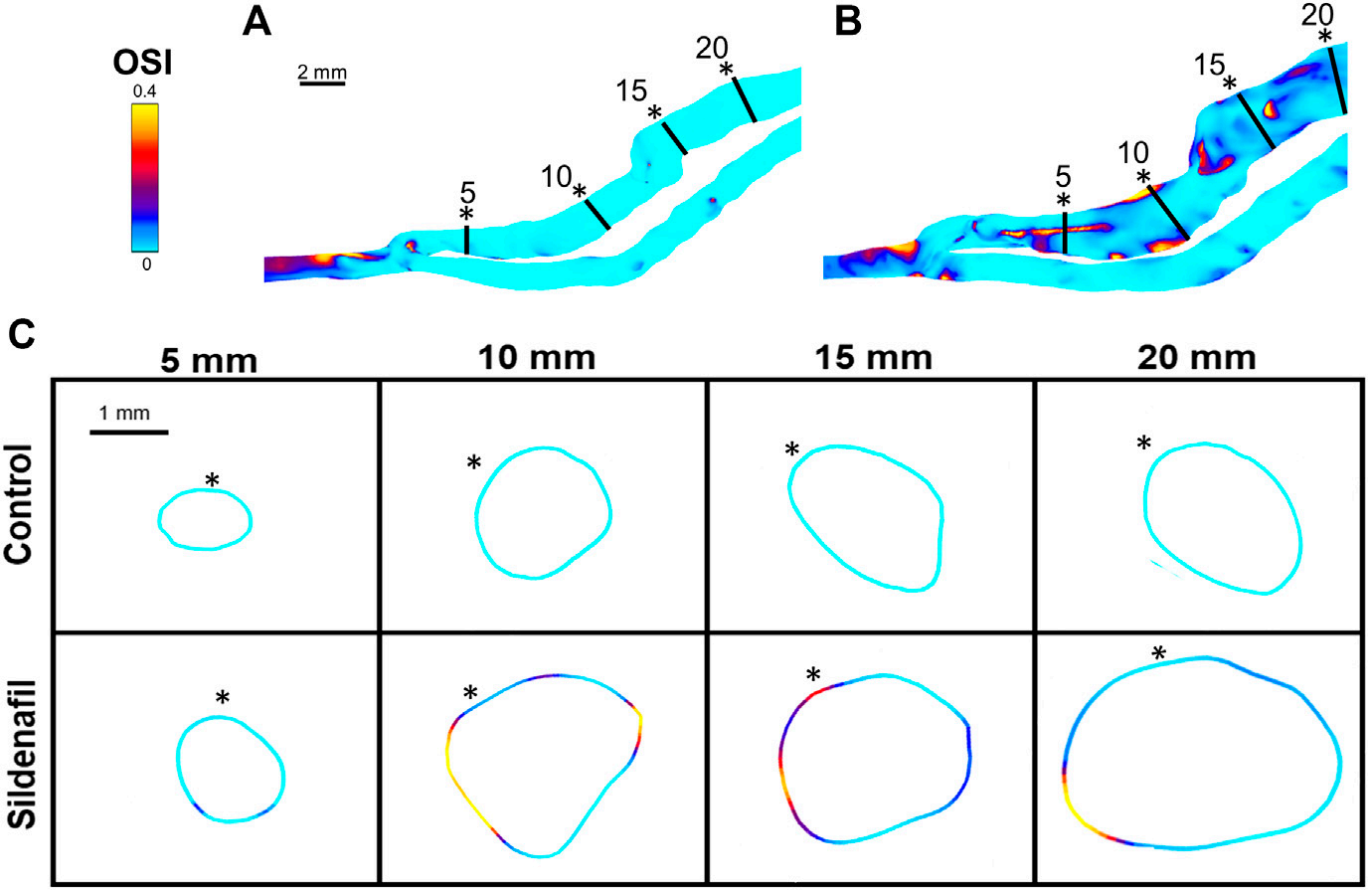
OSI color maps. **(A,B)** OSI for AVFs in a control rat **(A)** and a sildenafil treated rat **(B)**. **(C)** Color maps of OSI for control (top) and sildenafil (bottom) treated rats at 5, 10, 15, and 20 mm away from the anastomosis. Black lines in panels A and B represent the slice locations in panel C. * in panel C represents the top of the slice as shown in panels A and B. Color bar applies to **(A–C)**. Scale bar in A applies to A-B.

**FIGURE 8:**
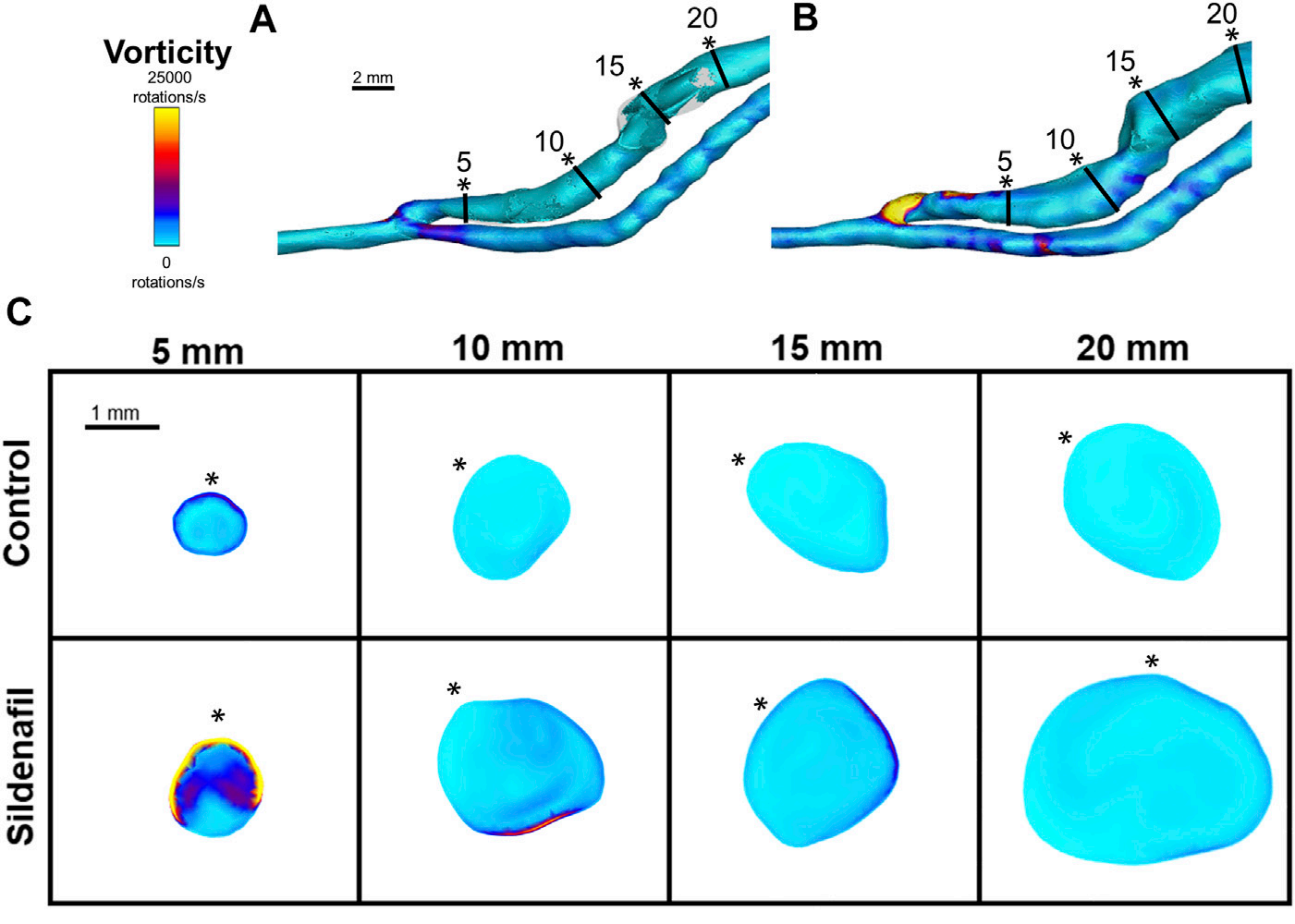
Vorticity isosurface and color maps. **(A,B)** Vorticity isosurface for AVFs in a control rat (A) and a sildenafil treated rat (B). **(C)** Color maps of vorticity for control and sildenafil treated rats at 5, 10, 15, and 20 mm away from the anastomosis. Black lines in panels A and B represent the slice locations. * in panel C represents the top of the slice as shown in panels A and B. Color bar applies to **(A–C)**. Scale bar in A applies to **(A–B)**.

When taking the entire fistula vein (0–20 mm) into account, the treatment of sildenafil makes a significant difference in the hemodynamics of AVFs. Averaged velocity was 47.43 ± 23.55 cm/s and 58.57 ± 40.14 cm/s for control and sildenafil treated rats, respectively (*p* < 0.05), and the average flow rate was 61.44 ± 2.79 ml/min and 88.00 ± 1.90 ml/min for control and sildenafil treated rats, respectively (*p* < 0.05). WSS averaged over the entire AVF was 167.5 ± 171.4 dyne/cm^2^, and 291.8 ± 430.2 dyne/cm^2^ for control and sildenafil treated rats, respectively (*p* < 0.05) and average OSI was 0.034 ± 0.021 and 0.13 ± 0.033 for control and sildenafil treated rats, respectively (*p* < 0.05). Vorticity was 2,322 ± 1968 rotations/s and 3,254 ± 3,484 rotations/s for control and sildenafil treated rats, respectively (*p* < 0.05). Color maps of the hemodynamics of representative rats are shown in Figure 5, 6, 7, 8, with the remaining rats shown in Supplementary Figure S5, S6.

Although the sildenafil treatment significantly increased the hemodynamic parameters in the entire AVF, it is important to point out that this increase varies regionally (Table 2). In the nearest anastomosis area (i.e., the 0–5 zone), sildenafil treated rats were significantly higher than controls for all hemodynamics parameters (velocity, flow rate, WSS, OSI, and vorticity) (*p* < 0.05); this occurred in the 10–15 zone as well. However, in the 5–10 zone, sildenafil treated rats were significantly higher than controls only for flow rate and OSI. For the 15–20 zone, sildenafil treated rats were significantly higher than controls for all hemodynamic parameters except vorticity (*p* < 0.05). Thus, the impact of sildenafil treatment on hemodynamic parameters varied with location along the fistula vein.

**TABLE 2:** Hemodynamic parameter results by zone. Data reported as mean ± SEM.

	Zone (mm)	0–5	5–10	10–15	15–20
Velocity (cm/s)	Control	83.23 ± 2.73	42.94 ± 0.277	33.65 ± 0.827	29.91 ± 0.349
	Sildenafil	118.3 ± 5.48	47.31 ± 1.01	36.92 ± 0.274	31.73 ± 0.205
WSS (dyne/cm^2^)	Control	377.1 ± 33.7	117.4 ± 2.57	106.8 ± 3.87	68.60 ± 2.74
	Sildenafil	806.8 ± 88.0	138.4 ± 3.11	140.2 ± 3.42	81.72 ± 1.43
OSI	Control	0.038 ± 0.003	0.049 ± 0.003	0.032 ± 0.002	0.018 ± 0.002
	Sildenafil	0.088 ± 0.005	0.16 ± 0.001	0.14 ± 0.002	0.13 ± 0.002
Vorticity (rotations/s)	Control	5,115 ± 301	1913 ± 25.5	1,314 ± 37.8	946.9 ± 11.4
	Sildenafil	8,237 ± 539	2,255 ± 63.0	1,480 ± 21.7	1,043 ± 14.1

Within both the control group and the sildenafil group, velocity was highest near the anastomosis in the 0–5 zone. It then sharply decreased to nearly 50% in the 5–10 zone and continued to decrease significantly but not as drastically (Figure 4B). Accordingly, the difference in velocity between the control group and the sildenafil group was the largest near the anastomosis in the 0–5 zone (42% increase, *p* < 0.05). The difference between the 2 groups became smaller when further away from the anastomosis. As seen in Figure 5C, velocity was also not uniform radially, and the distribution varies by zone. Additionally, the velocity vectors, as seen in Figure 5C, show an irregular and non-uniform flow pattern at each slice.

Within both the control group and the sildenafil group, the 0–5 zone had the highest WSS when compared to every other zone (Figure 4B, 6); WSS then decreased when more proximal to the heart. The biggest drop was between the 0–5 zone and the 5–10 zone (69% decrease for control and 83% decrease for sildenafil). The difference in WSS between the control group and the sildenafil group was the largest near the anastomosis in the 0–5 zone (114% increase by sildenafil, *p* < 0.05). The difference between the 2 groups became smaller when further away from the anastomosis. WSS is defined by shear stress at the wall (Eq. 6), so Figure 6 only shows the WSS at the wall. Like velocity, WSS is not uniform around the wall at a single slice (Figure 6C).

For the sildenafil group, OSI showed the largest increase between the 0–5 zone and the 5–10 zone (82% increase, *p* < 0.05). After that initial increase, OSI decreased further away from the anastomosis (Figures 4B, 7). The control group showed the same trend, although the initial increase between the 0–5 and 5–10 zone was not significant. The decrease between the 5–10 and 10–15 zones for both the control and sildenafil groups were significant (control: 35% decrease, *p* < 0.05, sildenafil: 12% decrease, *p* < 0.05). The decrease between the 10–15 and 15–20 zone was only significant for the control group (44% decrease, *p* < 0.05). Like WSS, OSI is defined by shear stress at the wall (Eq. 7), so Figure 7 only shows the OSI at the wall. Similar to velocity and WSS, OSI shows heterogeneity at each slice (Figure 7C).

Vorticity was highest near the anastomosis in the 0–5 zone within both the control group and the sildenafil group (Figure 4B, 8). It decreased sharply between the 0–5 zone and 5–10 zone (63% decrease for control, 73% decrease for sildenafil) and then continued to decrease though not as drastic. The difference in vorticity between the control group and the sildenafil group was the largest near the anastomosis in the 0–5 zone (61% increase by sildenafil, *p* < 0.05). The difference between the 2 groups became smaller when further away from the anastomosis. Vorticity is also heterogeneous at each slice (Figure 8). However, the maximum vorticity seems localized near the wall in every slice (Figure 8C).

## Discussion

Our study found that sildenafil treatment led to favorable remodeling in rat femoral AVFs, with increases in CSA and flow rate compared to controls, suggesting that sildenafil treatment may be an effective treatment to enhance AVF maturation. Furthermore, our study presents detailed protocols and spatial characterization of hemodynamics and geometry in a rat model of AVF development. We found that the effect size of sildenafil on AVF remodeling and the relationship between hemodynamics and AVF remodeling depends on location with respect to the longitudinal distance from the anastomosis.

Sildenafil was first marketed in 1998 for erectile dysfunction (Ghofrani et al., 2006), and since then, it has been approved as a treatment for other diseases, including pulmonary arterial hypertension (Klinger et al., 2019), Raynaud phenomenon (Fries et al., 2005), and high-altitude pulmonary edema (Luks et al., 2019). Patients most commonly take sildenafil orally, with some pulmonary arterial hypertension patients receiving the treatment intravenously. The standard dose for pulmonary hypertension patients is 20 mg thrice a day orally or 10 mg thrice a day intravenously. The maximum dose is 80 mg thrice a day orally. For Raynaud phenomenon, patients start with 20 mg once or twice daily and can increase up to 50 mg twice or thrice times daily (Sildenafil: Drug information—UpToDate). In 2019 a small pilot study focusing on safety was successfully completed for ESKD patients receiving AVF creation surgery. Patients received 20 mg twice daily starting at the first vascular test visit until 2 weeks post AVF creation surgery (Lee, 2018). Taken together, these studies showed the safety of sildenafil in ESKD patients and its efficacy in other non-ESKD diseases. Our rat study adds key discoveries in the application of sildenafil in promoting AVF development. We were able to safely deliver sildenafil to rats with AVFs, find sildenafil to improve AVF CSA and flow, and understand the detailed effects of sildenafil on AVF geometry and hemodynamics.

Lumen size is a combination of both outward remodeling (i.e., lumen expansion) and inward remodeling (i.e., neointimal hyperplasia formation). Our current MRI protocol gives a clear lumen image but does not detect neointimal hyperplasia. Therefore, the effect of sildenafil on neointimal hyperplasia is unknown in the present study due to the limitations of MRI. The smaller CSA near the anastomosis versus bigger CSA further downstream in rat AVFs may be attributed to neointimal hyperplasia near the anastomosis, the limited lumen expansion caused by the suture of the AVF, and/or the natural anatomy of the vasculature as native veins increase in CSA proximal to the heart. Regardless of the presence or absence of intimal hyperplasia, sildenafil significantly enhances CSA in rat AVFs, suggesting that sildenafil promotes outward remodeling. Future studies are warranted to investigate underlying molecular mechanisms.

Previous studies have shown that the configuration of the vein or arteriovenous anastomosis after AVF creation affects juxta anastomosis stenosis formation and maturation failure (Bharat et al., 2012; Sadaghianloo et al., 2015; Rezapour et al., 2018). Bharat et al. used the piggyback straight-line onlay technique (pSLOT) to create AVFs with the vein being in a straight line, “piggybacking” on the artery at the site of anastomosis, allowing for the arterial blood to flow straight into the cylindrical lumen. They found that pSLOT patients had significantly less juxta anastomosis stenosis than controls (Bharat et al., 2012). Sadaghianloo et al. used an angled ruler and protractor to measure the anastomosis angle of patients who underwent AVF creation surgery. Anastomosis angle of <30° for radiocephalic fistulas were associated with reduced primary and secondary patency (Sadaghianloo et al., 2015). On the other hand, Yang et al. reported that an anastomotic angle wider than 46.5° might lead to disturbed flow generation in radio-cephalic AVFs (Yang et al., 2020). Hull et al. used idealized models of side to side and end to side anastomosis for CFD simulations. They found that the side to side configuration as well as the end to side at 90° had higher venous outflow than end to side 45° configuration (Hull et al., 2013). Thus, while anastomosis angles affect AVF blood flow and maturation, the optimal angle still remains unclear.

Centerline-based approaches for geometric analysis of blood vessels have been reported in the literature (Kamenskiy et al., 2012; Aristokleous et al., 2018; Corbett et al., 2018). Therefore, our group used this approach to characterize AVFs in human (He et al., 2021) and mouse (Falzon et al., 2020), and here we used it on rats. Previously we reported that carotid jugular AVFs created in NOS3 overexpression mice had bigger CSA and flow than AVFs created in NOS3 knockout mice and wild-type mice (Pike et al., 2019; Falzon et al., 2020), but AVFs in three mouse strains had similar anastomosis angle, tortuosity, and nonplanarity angle (Falzon et al., 2020). Likewise, in patients, the anastomosis angle, tortuosity, and nonplanarity angle were not associated with AVF maturation (He et al., 2021). Here we found that anastomosis angle, tortuosity, and nonplanarity angle magnitude were all similar between control AVFs and sildenafil treated AVFs, even though the latter have bigger CSA and flow rates. It is possible that the sildenafil treatment does not affect the centerline of the fistula vein. It is also possible that centerline-based methods may not adequately describe the complex geometry in AVFs. Different approaches for geometric analysis, such as statistical shape analysis, may be considered to investigate how geometric parameters affect AVF remodeling in future studies.

Hemodynamics are generally accepted to be a critical cause and regulator of vascular remodeling. The elevated blood flow rate in the vein caused by AVF creation results in increased WSS. When endothelial cells are exposed to non-physiological WSS, they produce molecules signaling for multiple pathways, including smooth muscle cell proliferation (Cunnane et al., 2017; Remuzzi and Bozzetto, 2017), which can lead to neointimal hyperplasia development (Cunnane et al., 2017). It has also been found that OSI and multidirectional flow occur at AVF locations prone to stenosis (Cunnane et al., 2017). Consequently, we analyzed the hemodynamic parameters of flow rate, velocity, WSS, OSI, and vorticity. We found that volumetric flow rate is constant in our region of interest, as the main AVF segment of analysis has not branched, and hence velocity is reversely related to area. As the CSA gradually increase when further away from the AV anastomosis, velocity has the highest values in the 0–5 zone, a drastic decrease between the 0–5 and 5–10 zones, and then decreases through the rest of the zones. WSS and vorticity also follow the same pattern. The drastic decrease in velocity, WSS, and vorticity away from the anastomosis could be explained by a pressure drop. Browne et al. showed a significant and quick pressure drop at the anastomosis in patients *in vivo* and through CFD (Browne et al., 2015b), which is also the location where the velocity, WSS, and vorticity dropped in our rat AVF model. Future studies can measure pressure in rats to further investigate the relationship between pressure and hemodynamics.

Our previous studies showed a return to baseline WSS levels in AVFs created in NOS3 overexpression mice, but not in NOS3 knockout mice or wild-type mice, in 21 days after AVF creation (Pike et al., 2019). This return to baseline WSS levels has been reported in other AVF literature and is often explained by the vessel’s tendency to normalize WSS through remodeling after AVF creation. Readers are referred to excellent review papers on this topic (Rothuizen et al., 2013; Browne et al., 2015a). However, we did not see a return of WSS to baseline in our rat model. Possible explanations for this difference include the differences in animal models (mouse vs rat) and in mechanisms (NOS3 vs PDE5A). In particular, we observed an increased cardiac output in the sildenafil treated rats [data not shown], which could explain the sustained increase in flow, and hence velocity and WSS.

OSI is often reported in literature to be localized in areas prone to vascular stenosis (Cunnane et al., 2017). In arterial literature, high OSI is often used as a marker of disturbed flow and is associated with atherosclerotic lesions (Peiffer et al., 2013). However, our results show that sildenafil treated rats had higher OSI in AVFs than control AVFs while still having better AVF outcomes. It is possible that there may be factors that are more dominant than OSI in influencing AVF remodeling, such as WSS. In addition, all levels of OSI in our rat AVFs were under 0.2. OSI higher than 0.2 has been used in literature to mark high (vs low) OSI and is associated with increased risk of atherosclerosis; therefore, while OSI is different between the treatment groups and zones along the rat AVF, it may not be high enough to affect AVF remodeling.

Our study has several strengths. Our study uses sildenafil which has been FDA approved for multiple uses for several years. Thus, the transition from bench to bedside for sildenafil use to promote AVF maturation can be smooth as safety is not a concern. Another strength is the applicability of our analysis technique to patients. Contrast agents that are often used in MRI procedures are known to lead to toxicity in patients with impaired kidney function. Our method, however, is contrast-agent free, so every parameter and analysis performed in this study can be applied to patients. Additionally, our study used live animals and non-invasive methods to observe the lumen of a blood vessel filled with blood, which provides accurate lumen size that cannot be achieved by histology and 2D ultrasound. Another benefit of our study is that by using live animals and MRI, we were able to provide a 3D *in situ* model with realistic AVF configuration and real-time blood velocity, whereas the majority of CFD analyses of AVFs use idealized parameters.

Limitations of the study include our focus on one time point. Extending our study to include a shorter or longer time point in future studies may be needed to better understand the temporal mechanisms and efficacy of sildenafil. However, studies have shown that in the first few weeks after AVF creation, the blood flow and diameter increase are predictive of AVF maturation (Robbin et al., 2018). This study did not investigate histology; in the future, we can consider including histology to investigate whether locations with smaller lumen area indeed have hyperplasia. We used a systemic treatment that may have effects beyond the AVF; for example, sildenafil treated rats have increased heart output [data not shown]. Future studies may consider localized delivery of sildenafil. A previous clinical study reported that the effect size of sildenafil on patients with pulmonary arterial hypertension varies with doses (Galie et al., 2005). Our study used only one dose (90 mg), and future studies may consider different doses. Another limitation is that our study was performed in healthy rats. Patients with AVFs have CKD and are known to have impaired vascular functions (Allon et al., 2016; Dember et al., 2016). Thus, future studies should consider the use of CKD animals to investigate the efficacy of sildenafil in AVF development. Lastly, this study used a rigid wall assumption to both sildenafil-treat rats and control rats. Future studies may consider to measure the biomechanical properties of the vessel walls (for example, Young’s Modulus) and use them in order to 1) understand any effect of sildenafil on the vascular wall’s mechanical properties and 2) perform fluid structure interaction (FSI) simulations that treat blood vessel walls as deformable.

## Conclusion

The animals tolerated sildenafil well and sildenafil increased the AVF lumen cross-sectional area and flow, which are important clinical outcomes of AVFs. We also analyzed the effect of sildenafil on detailed hemodynamics and geometry in rat femoral AVFs using MRI based CFD and geometric analysis. We found that the magnitudes of hemodynamics parameters, including velocity, WSS, OSI, and vorticity, were heterogeneous along the AVF, which indicates location dependence of the effect size of sildenafil on AVF remodeling and the relationship between hemodynamics and AVF remodeling. In particular, velocity, WSS and vorticity were all highest near the anastomosis, the location where stenosis is common, and then dropped significantly further away. Overall, our results suggest that sildenafil has the potential as an effective therapy to improve AVF remodeling by increasing cross-sectional area and flow rate, and that the heterogeneous nature of the AVF must be considered when analyzing treatment effect size and investigating underlying mechanisms. These exciting findings pave the way for future studies that can expand and consider dosing and local delivery of sildenafil in animals with CKD, biomechanical properties of the wall, and temporal analysis of hemodynamic changes using FSI.

## Data Availability

The original contributions presented in the study are included in the article/Supplementary Material, further inquiries can be directed to the corresponding authors.
