# Supplementary figures and images for: Analysis of Geometric and Hemodynamic Profiles in Rat Arteriovenous Fistula Following PDE5A Inhibition
Source: Front Bioeng Biotechnol. 2021 Dec 2;9:779043. doi: 10.3389/fbioe.2021.779043 (PMC8675087; doi:10.3389/fbioe.2021.779043)

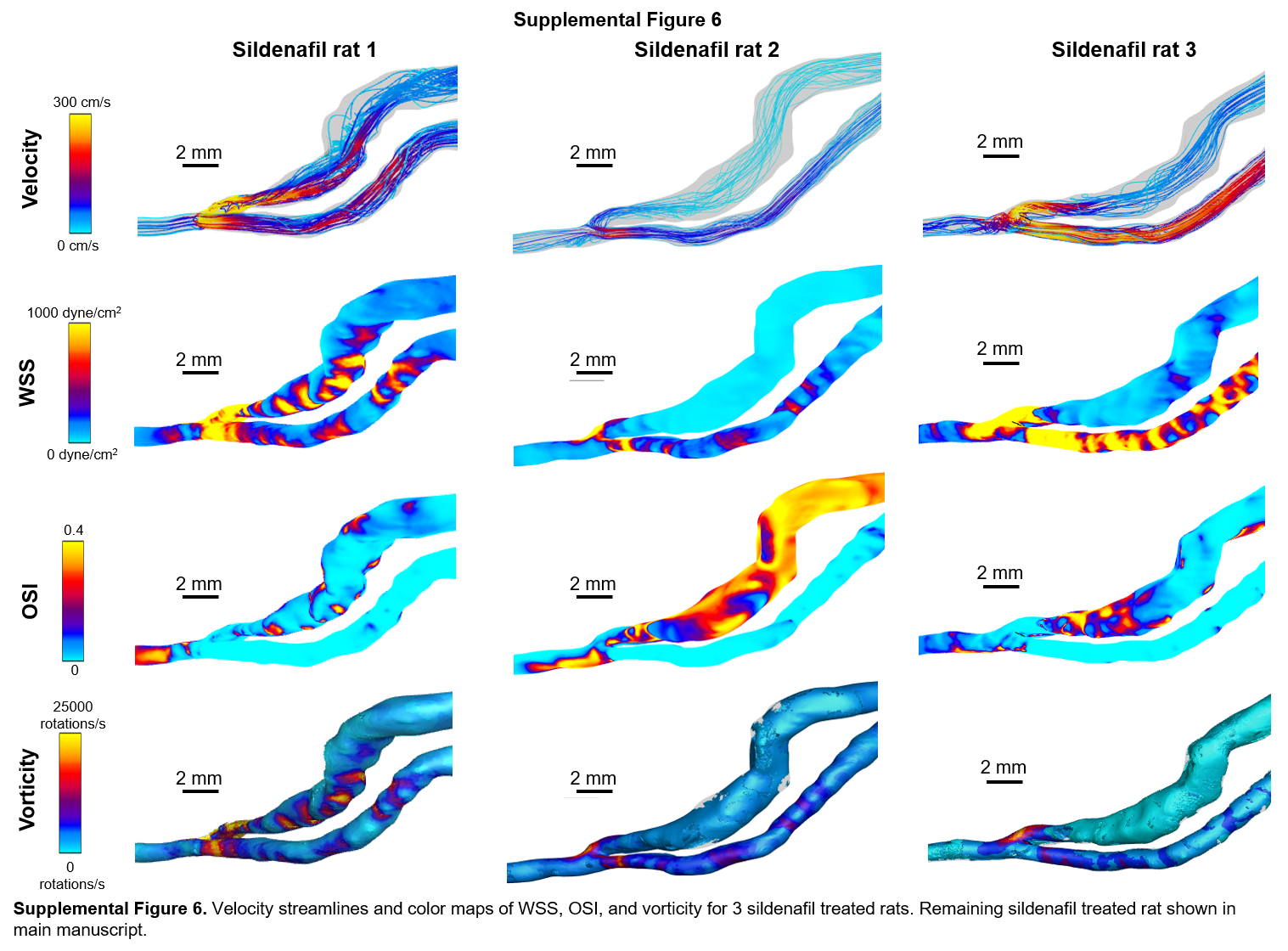

Supplement: Supplementary file 1 [file Image6.tif]

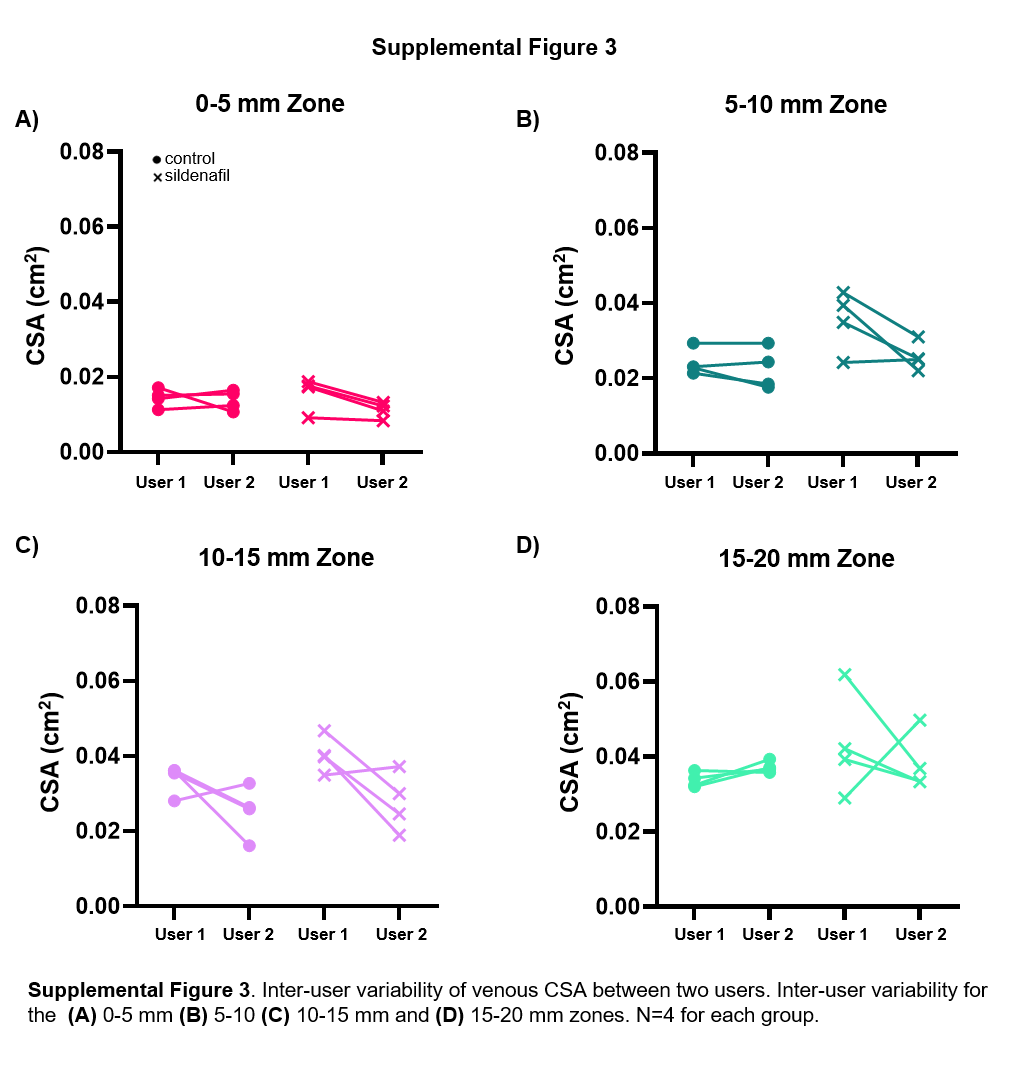

Supplement: Supplementary file 2 [file Image3.tif]

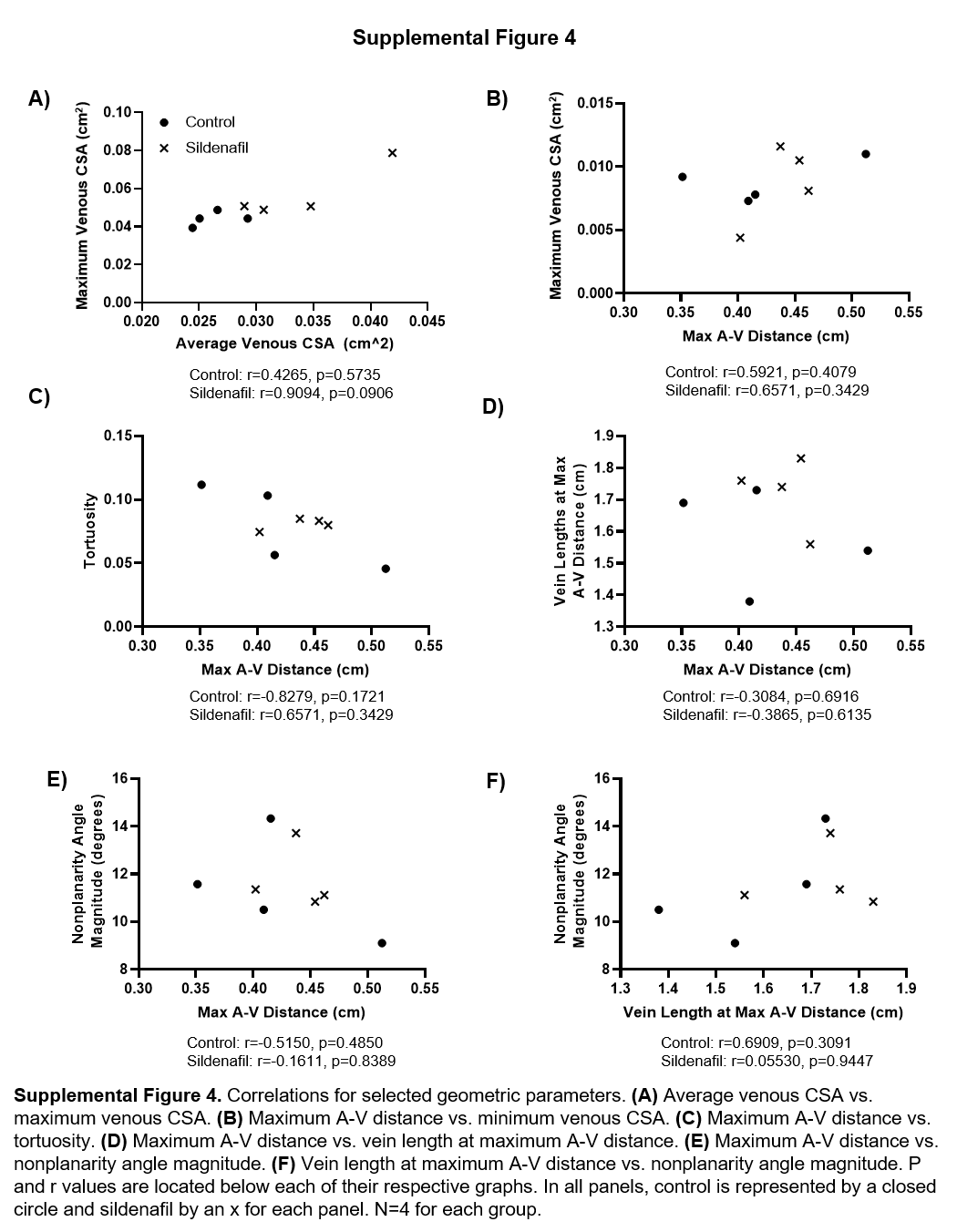

Supplement: Supplementary file 3 [file Image4.tif]

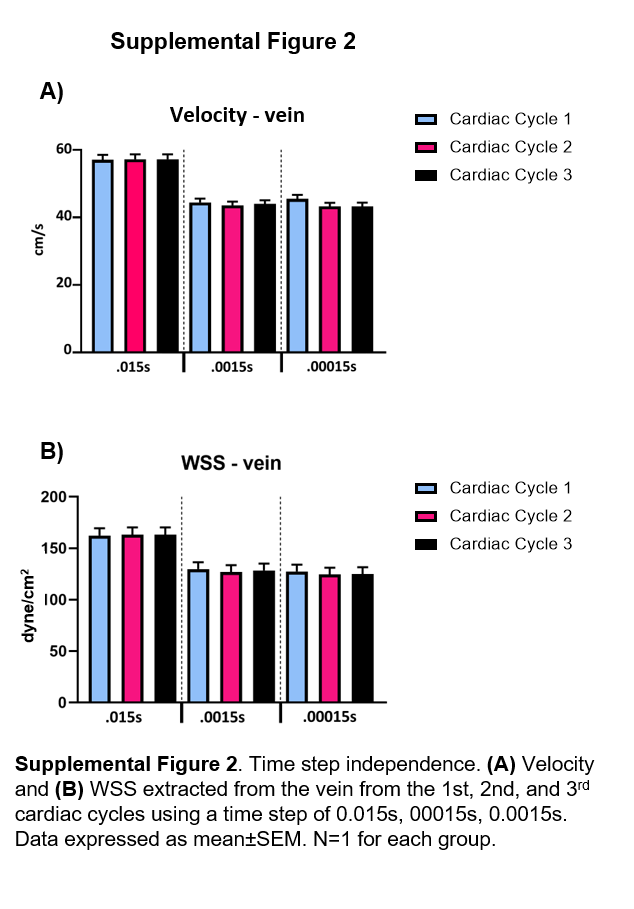

Supplement: Supplementary file 4 [file Image2.tif]

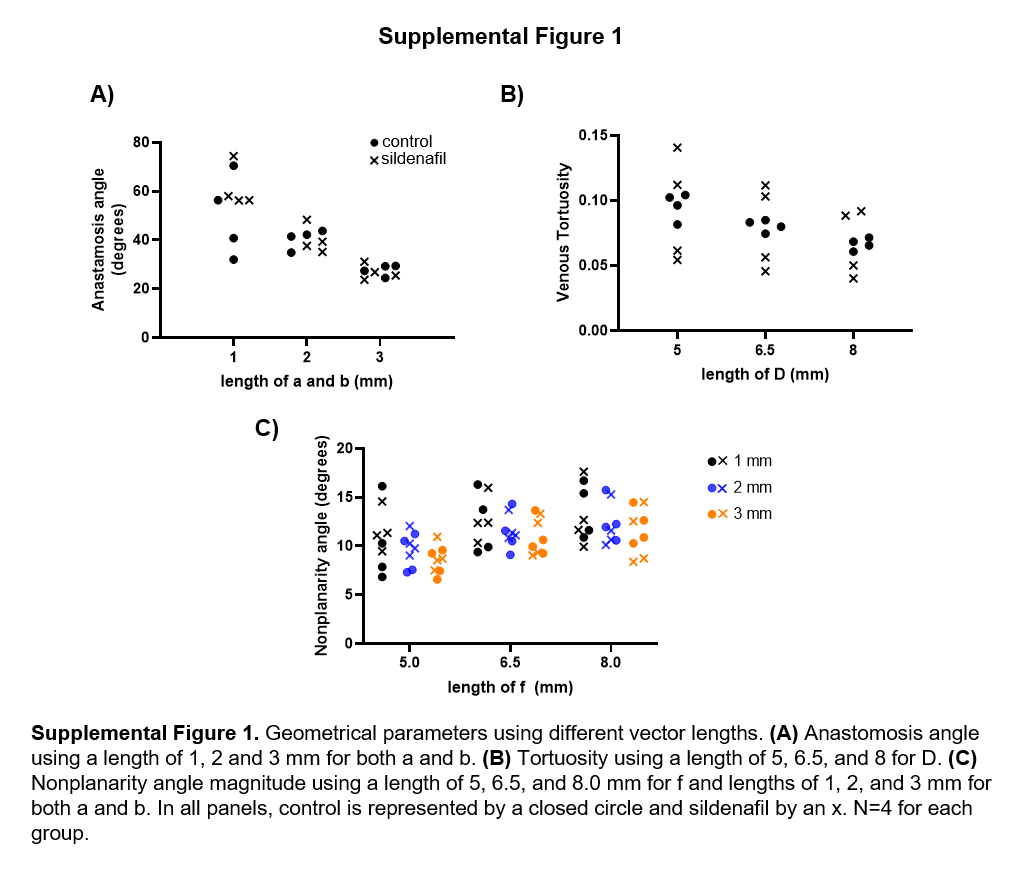

Supplement: Supplementary file 5 [file Image1.tif]

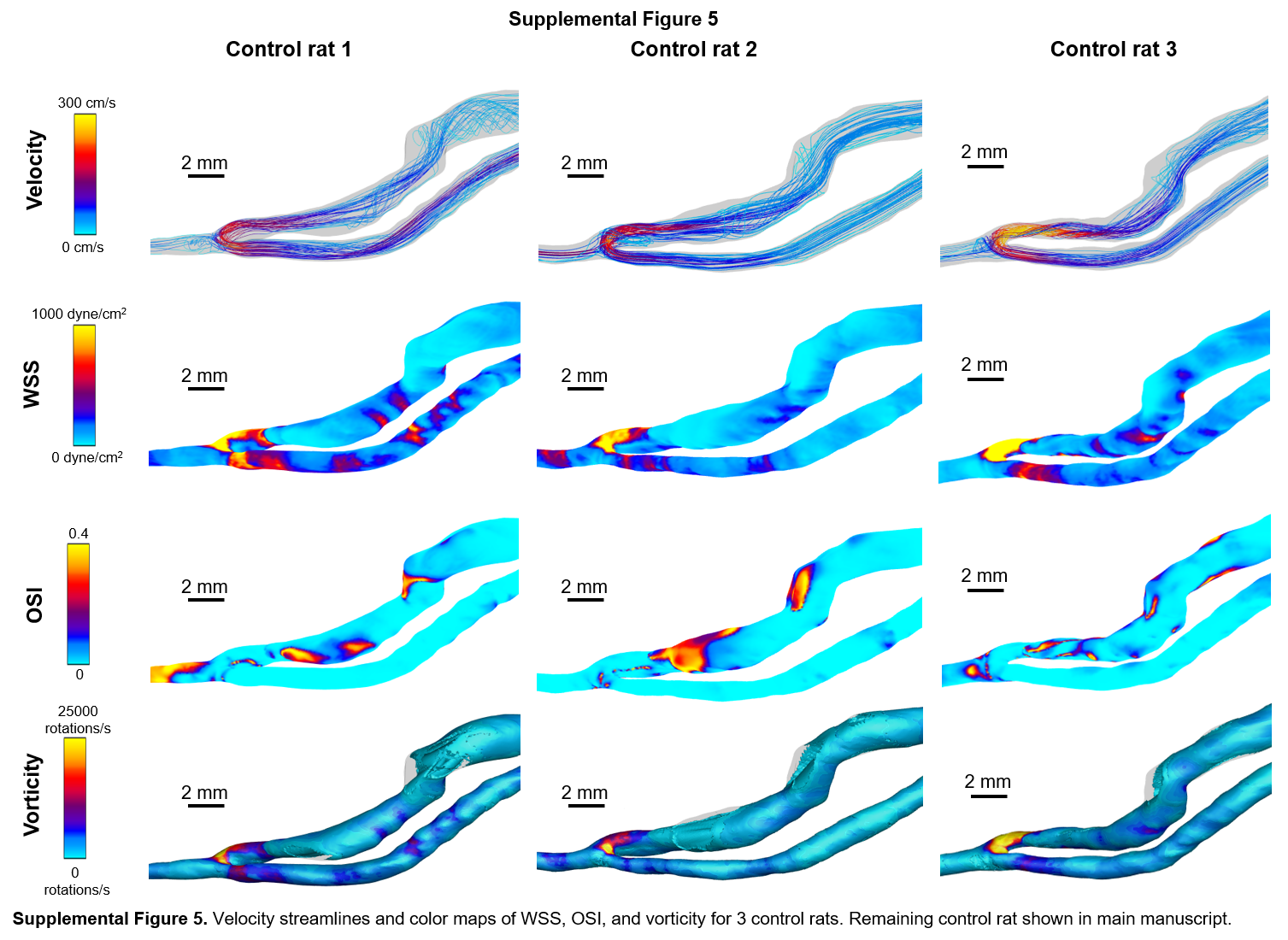

Supplement: Supplementary file 6 [file Image5.tif]
